# Radiotherapy quality assurance for mesorectum treatment planning within the multi-center phase II STAR-TReC trial: Dutch results

**DOI:** 10.1186/s13014-020-01487-6

**Published:** 2020-02-18

**Authors:** Roy P. J. van den Ende, Femke P. Peters, Ernst Harderwijk, Heidi Rütten, Liza Bouwmans, Maaike Berbee, Richard A. M. Canters, Georgiana Stoian, Kim Compagner, Tom Rozema, Mariska de Smet, Martijn P. W. Intven, Rob H. N. Tijssen, Jacqueline Theuws, Paul van Haaren, Baukelien van Triest, Dave Eekhout, Corrie A. M. Marijnen, Uulke A. van der Heide, Ellen M. Kerkhof

**Affiliations:** 1 Department of Radiation Oncology, Leiden University Medical Center, P.O. Box 9600 2300, RC, Leiden, the Netherlands; 2 Department of Radiation Oncology, The Netherlands Cancer Institute, Amsterdam, the Netherlands; 3 Department of Radiation Oncology, Radboud University Medical Center, Nijmegen, the Netherlands; 4 Department of Radiation Oncology, Maastricht University Medical Center, Maastricht, the Netherlands; 5 Department of Radiation Oncology, Isala Clinics, Zwolle, the Netherlands; 6 Department of Radiation Oncology, Verbeeten Institute, Tilburg, the Netherlands; 7 Department of Radiotherapy, University Medical Center Utrecht, Utrecht, the Netherlands; 8 Department of Radiation Oncology, Catharina Hospital, Eindhoven, the Netherlands

**Keywords:** Rectal neoplasms, Radiotherapy, Treatment planning, Quality assurance

## Abstract

**Background:**

The STAR-TReC trial is an international multi-center, randomized, phase II study assessing the feasibility of short-course radiotherapy or long-course chemoradiotherapy as an alternative to total mesorectal excision surgery. A new target volume is used for both (chemo)radiotherapy arms which includes only the mesorectum. The treatment planning QA revealed substantial variation in dose to organs at risk (OAR) between centers. Therefore, the aim of this study was to determine the treatment plan variability in terms of dose to OAR and assess the effect of a national study group meeting on the quality and variability of treatment plans for mesorectum-only planning for rectal cancer.

**Methods:**

Eight centers produced 25 × 2 Gy treatment plans for five cases. The OAR were the bowel cavity, bladder and femoral heads. A study group meeting for the participating centers was organized to discuss the planning results. At the meeting, the values of the treatment plan DVH parameters were distributed among centers so that results could be compared. Subsequently, the centers were invited to perform replanning if they considered this to be necessary.

**Results:**

All treatment plans, both initial planning and replanning, fulfilled the target constraints. Dose to OAR varied considerably for the initial planning, especially for dose levels below 20 Gy, indicating that there was room for trade-offs between the defined OAR. Five centers performed replanning for all cases. One center did not perform replanning at all and two centers performed replanning on two and three cases, respectively. On average, replanning reduced the bowel cavity V20Gy by 12.6%, bowel cavity V10Gy by 22.0%, bladder V35Gy by 14.7% and bladder V10Gy by 10.8%. In 26/30 replanned cases the V10Gy of both the bowel cavity and bladder was lower, indicating an overall lower dose to these OAR instead of a different trade-off. In addition, the bowel cavity V10Gy and V20Gy showed more similarity between centers.

**Conclusions:**

Dose to OAR varied considerably between centers, especially for dose levels below 20 Gy. The study group meeting and the distribution of the initial planning results among centers resulted in lower dose to the defined OAR and reduced variability between centers after replanning.

**Trial registration:**

The STAR-TReC trial, ClinicalTrials.gov Identifier: NCT02945566. Registered 26 October 2016, https://clinicaltrials.gov/ct2/show/NCT02945566).

## Background

Only 2% of the patients with early stage rectal cancer treated with total mesorectal excision (TME) surgery experience local failure and 12% develop a distant failure [1–3]. However, TME surgery can result in significant morbidity and mortality [4–6]. For a distal tumor, approximately 40% of patients require a permanent stoma. Complications of TME surgery include anastomotic leaks, urinary and fecal incontinence and sexual dysfunction. Therefore, research for this early stage rectal cancer patient group has focused on alternative strategies, such as limited resections and active surveillance of good responders after chemoradiotherapy [7–10].

The STAR-TReC trial is an international multi-center, randomized, phase II study assessing the feasibility of short-course radiotherapy (SC-RT) or long-course chemoradiotherapy (LC-CRT) with subsequent two-stage response assessment as an alternative to TME surgery. Patients are randomized between; a) TME b) organ preservation utilizing LC-CRT and c) organ preservation utilizing SC-RT (ClinicalTrials.gov Identifier: NCT02945566) [11]. A novel target volume is used which includes only the mesorectum [12].

Before patient accrual, each center had to go through a radiotherapy quality assurance (QA) program led by national radiotherapy trial teams, including a delineation and a treatment planning case. The results for the treatment planning showed substantial variation in dose to organs at risk (OAR), suggesting that different trade-offs were made in each center. Therefore, the Dutch radiotherapy trial team (FP, CM and EK) decided to extend the QA program with four additional planning cases and organized a study group meeting for the Dutch centers in the STAR-TReC trial. The aim of this study was to determine the variability in treatment plans in terms of dose to OAR and to determine the effect of a study group meeting on the quality and variability of treatment plans for mesorectum-only planning for rectal cancer.

## Methods

### Participating centers and patients

Eight centers participated in this study. We selected 5 cases, including the treatment planning case of the radiotherapy QA program, according to the STAR-TReC inclusion criteria with a small (< 4 cm) T1-3bN0M0 tumor without involvement of the mesorectal fascia or extra-mural vascular invasion.

### Target volume and OAR

The STAR-TReC trial utilizes an adapted definition of the clinical target volume (CTV) that only includes the mesorectum from two centimeters below the tumor up to the S2–3 interspace level. This is a smaller CTV compared to the current standard for radiotherapy of rectal cancer and it is specially tailored for early stage disease, with the goal of organ preservation. The development of this new mesorectal CTV definition was described in Peters et al. [12]. The planning target volume (PTV) was defined as the CTV plus a margin of 15 mm in the anterior direction and 10 mm in the posterior, lateral and craniocaudal directions. The defined OAR were the bowel cavity, bladder and femoral heads. The bowel cavity was delineated using adapted RTOG guidelines, including abdominal contents and excluding major vasculature, muscles and bones, other pelvic organs (e.g. bladder, prostate, vagina, uterus) and the CTV. The bowel cavity volume was delineated up to 2 cm above the superior extent of the PTV and inferiorly where small bowel or colon was visible. The whole bladder was delineated including the bladder wall. The femoral heads were delineated to the most inferior extent of the lesser trochanter. The CTV and the OAR of all 5 cases were delineated by one observer (FP) on the planning CT scan.

### Treatment planning

We sent the planning CT scans with the corresponding delineations to each participating center, so that each center performed treatment planning based on the same delineations. They were asked to produce LC-CRT treatment plans of 25 × 2 Gy according to the STAR-TReC study protocol. The participating centers were experienced centers in the treatment of rectal cancer patients. Different planning systems and treatment delivery techniques were used among the centers, as shown in Table 1. Treatment plans were produced by radiotherapy technologists with 0–20 years of treatment planning experience. Participating centers used the same criteria regarding delivery efficiency as they would use in clinical practice, the constraints regarding delivery efficiency are shown in Table 1.

**Table 1:** Participating centers with corresponding planning parameters

Institute	TPS	Technique	Energy (MV)	Number of arcs/fields	Number of arcs/fields	Delivery efficiency	RTT experience (years)	Automated treatment planning
**1**	Pinnacle	AT	10	Dual full arc	2	Maximum delivery time 60 s	11	Yes, Pinnacle AutoPlanning^b^
**2**	Pinnacle	AT	6	One or two full arcs^a^	1 or 2^a^	Maximum delivery time 60–90 s per arc	15	No
**3**	Pinnacle	AT	10	Two dual partial arcs	4	Maximum delivery time 160 s	0–20	Yes, Pinnacle AutoPlanning
**4**	Pinnacle	AT	10	Two dual partial arcs	4	Maximum delivery time 100 s	5	No
**5**	Eclipse	AT	10	Two full arcs	2	Maximum 3x prescribed dose in cGy for MU	10	No
**6**	Eclipse	AT	10	Two full arcs	2	Maximum 3x prescribed dose in cGy for MU	20	Yes, RapidPlan
**7**	Monaco	AT	10	Two partial arcs	2	Maximum 4x prescribed dose in cGy for MU	11	No
**8**	RayStation	IMRT	10	Five fields	5	Maximum 15 segments. Minimal segment area 35 cm^2^, minimal 4 MU/segment	12	No

*TPS* treatment planning system, *AT* Arc Therapy, *IMRT* Intensity Modulated Radiotherapy, *MU* monitor units, *RTT* Radiotherapy Technologist

^a^Depending on patient anatomy, ^b^Only for the replanning

The target volume constraints were defined according to the ICRU 83 criteria, focusing on full coverage of the target volume with CTV V95% = 100%, PTV V95% ≥ 99%, PTV V90% = 100%, PTV V105% ≤ 1 and 98% ≤ PTV D50% ≤ 102%. There is lack of data on the association of dose to bowel, bladder and femoral heads and the risk of late complications for dose levels up to 50 Gy. Therefore, the study protocol had no mandated OAR constraints but specified optimization objectives for the OAR adapted from Appelt et al. [13]: bowel cavity V20Gy < 190 cc, V30Gy < 130 cc, V45Gy < 100 cc, bladder V35Gy < 22%, V50Gy < 7% and femoral head left and right V25Gy < 14%.

### Study group meeting

After all centers had completed the treatment planning on all cases, we organized a study group meeting to discuss the planning results. A radiation-oncologist, a medical physicist and a radiotherapy technologist with rectal cancer expertise of each participating center were invited for this meeting. During the meeting, we visualized the values of the DVH parameters of all treatment plans and the dose distributions of specific cases to discuss the differences.

We distributed the values of the DVH parameters of all treatment plans among all centers so that results could be compared. Subsequently, we invited the participating centers to perform replanning if they considered this to be necessary.

### Treatment plan comparison

Each participating center returned for each case the DVH parameters and the dose distribution of the initial planning and replanning in DICOM format. To avoid differences in DVH parameters due to different treatment planning systems, we calculated the values of the DVH parameters using an independent DVH calculation on the submitted dose distributions. We assessed the accuracy of our independent DVH calculation algorithm using a gamma-analysis between dose volume histograms calculated by the algorithm and dose volume histograms calculated by our treatment planning system for a dataset of eight patients with varying target volumes and organs at risk. We used a tolerance of 0.1 Gy and 1% volume. In addition, we compared the DVH parameters calculated by the algorithm with the DVH parameters submitted by the participating centers, which were calculated by their treatment planning systems.

To determine the effect of the study group meeting on the dose to OAR, we compared the values of the DVH parameters of the replanned cases to the initial plans. In addition to the DVH parameters of the OAR optimization objectives, we selected additional DVH parameters for a more detailed evaluation of the differences in low dose levels. The additional parameters included the V5Gy, V10Gy, and V15Gy for the bowel cavity as well as the bladder.

No constraints or objectives were imposed on the additional DVH parameters.

To investigate the total volume of the V10Gy and V25Gy, we added a volume that included the patient contour of the planning CT scan. In addition, we added a volume that included the patient contour of the planning CT scan but excluded the PTV, bowel cavity, bladder and femoral heads to investigate if the dose to other normal tissue was increased while sparing the defined OAR. This volume is called “Non-defined OAR”.

For the cases for which no replanning was performed, we reported the values of the DVH parameters of the initial planning of those centers as the result of the replanning.

### Statistical analysis

We used SPSS Statistics 23 (IBM Corp. Released 2015. IBM SPSS Statistics for Windows, Version 23.0. Armonk, NY: IBM Corp.) for statistical analysis. A paired samples T-test was used to test for differences in the values of the DVH parameters between the initial planning and replanning. The significance threshold was set at *p* < 0.002, adjusted for multiple testing using the Bonferroni correction.

## Results

### DVH calculation algorithm

The gamma passing rate of the dose volume histograms calculated by the independent DVH calculation algorithm was 99.99 ± 0.03%. On average, the relative difference between the DVH parameters calculated by the independent DVH calculation algorithm and the DVH parameters submitted by the centers was − 0.5 ± 0.2%.

### Initial planning

**Fig. 1:**
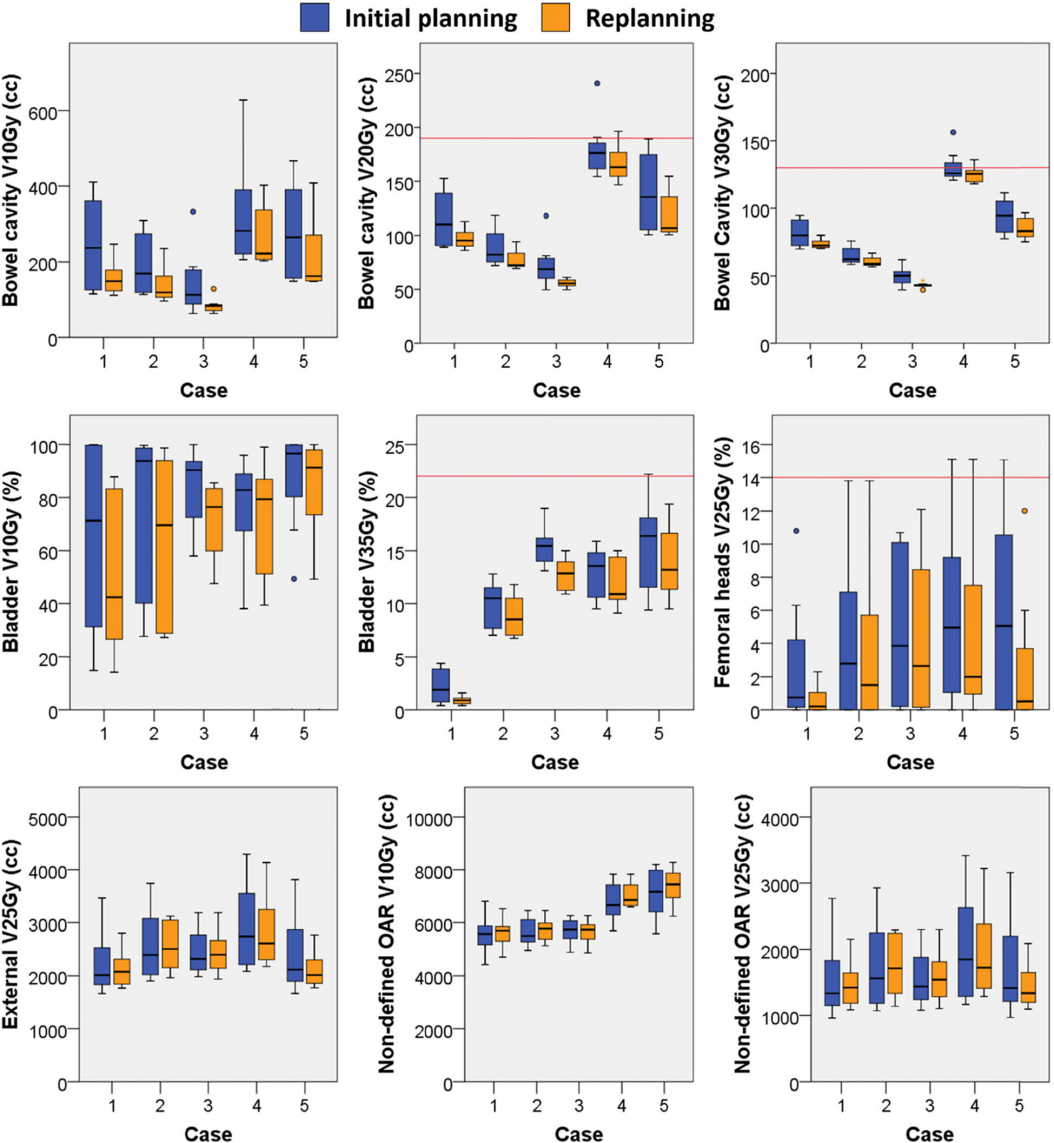
Planning results for the initial planning (blue) and replanning (orange) for each case. The red lines indicate the OAR optimization objective

All plans satisfied the target volume constraints. Large differences in dose to OAR were observed, especially in the dose levels below 20 Gy, as shown in Fig. 1. The differences were discussed at the study group meeting, where it was concluded that the variation was mostly due to differences in local practice and lack of evidence for OAR constraints. As there is insufficient evidence to support prioritizing the sparing of OAR at these low dose levels, the prioritization was left to the center’s preference.

### Replanning

Five centers performed replanning for all cases. Center 2 performed replanning on two cases, center 3 did not perform replanning at all and center 5 performed replanning on three cases. Center 1 used Pinnacle AutoPlanning for the replanning. All other centers performed replanning using the same treatment technique as the initial planning, as described in Table 1. All 30 replanned cases fulfilled the target volume constraints. For all cases, replanning resulted on average in a lower dose to the defined OAR (Table 2). The bowel cavity V5Gy, V10Gy, V15Gy, V20Gy, V30Gy and V45Gy and the bladder V10Gy, V15Gy and V35Gy were significantly lower in the replanning for all cases (*p* < 0.001). On average, replanning reduced the bowel cavity V20Gy by 12.6%, the bowel cavity V10Gy by 22.0%, the bladder V35Gy by 14.7% and the bladder V10Gy by 10.8%.

**Table 2:** Absolute difference between replanning and initial planning. A positive difference means a higher value for the replanning. All values are presented as mean (range)

	Case 1	Case 2	Case 3	Case 4	Case 5
CTV V95 (%)	0.0 (0.0–0.0)	0.0 (0.0–0.0)	0.0 (0.0–0.0)	0.0 (0.0–0.0)	0.0 (0.0–0.0)
PTV V95 (%)	− 0.3 (− 0.9–0.2)	− 0.1 (− 0.3–0.0)	− 0.2 (− 0.7–0.0)	−0.1 (− 0.3–0.0)	−0.1 (− 0.3–0.0)
PTV V90 (%)	0.0 (0.0–0.0)	0.0 (− 0.1–0.0)	0.0 (−0.1–0.0)	0.0 (0.0–0.0)	0.0 (− 0.1–0.0)
Bowel cavity volume (cc)	992.0	754.4	721.3	1306.3	1273.2
Bowel cavity V5Gy (cc)	− 120.9 (− 371.9–0)	− 78.9 (− 317.1–22.9)	− 106.2 (− 299.6–0)	− 107.8 (− 426.1–44.5)	− 141.5 (− 529.2–0)
Bowel cavity V10Gy (cc)	−89 (− 237.1–23.6)	− 55.2 (− 176.8–0)	−60.2 (− 203.6–0)	− 60.9 (− 226.1–0)	− 64.8 (− 198.8–0)
Bowel cavity V15Gy (cc)	−32.4 (− 68.5–5.2)	−23.5 (− 92.5–0)	− 28.4 (− 106.1–0)	− 22.7 (− 66.4–0)	− 33.9 (− 105.5–0)
Bowel cavity V20Gy (cc)	−18.2 (− 46.7–6.7)	−11.3 (− 45.8–0)	−17.2 (− 61.4–0)	− 13.8 (− 44.6–0)	− 21.6 (− 58.4–0.1)
Bowel cavity V30Gy (cc)	−7.9 (− 22–6.8)	−4.6 (− 18.3–0)	−6.9 (− 19.6–0)	− 5.4 (− 20.3–0.8)	−9 (− 25–0)
Bowel cavity V45Gy (cc)	−3.2 (− 9.1–4.5)	−1.6 (− 6.5–0)	− 2.2 (− 6.8–0)	−1.4 (− 8.6–1.8)	−3.6 (− 8.7–0)
Bladder volume (cc)	197.2	344.7	232.3	277.3	89.2
Bladder V5Gy (%)	−6.5 (−21.4–0)	−6.4 (− 28.7–0)	−4.2 (− 16.1–0)	−5.6 (− 34.6–0)	0.1 (− 1.1–2.2)
Bladder V10Gy (%)	−14.2 (− 58.4–0)	− 10.3 (− 48.8–0)	− 12.3 (− 45.5–0)	−4.9 (− 32.6–20)	−3.5 (− 17–0.2)
Bladder V15Gy (%)	−14.8 (− 59.2–0)	− 7.5 (− 33.5–1.1)	−13.1 (− 33.6–0)	− 6.1 (− 22.2–1)	− 3.4 (− 13.8–0.1)
Bladder V35Gy (%)	−1.3 (−3.4–0.2)	−1.1 (− 5–0.5)	−2.7 (− 7.4–0)	−1 (− 3.7–0)	−1.6 (− 10.7–3.3)
Bladder V50Gy (%)	0 (0–0)	0.1 (− 0.2–0.7)	0.3 (−1–1.8)	0.3 (− 0.6–1.5)	0.1 (−1.3–0.8)
External volume (cc)	27,105.6	21,073.2	18,660.9	23,076.3	28,088.9
External_V10Gy_(cc)	−61.9 (− 645.1–645.2)	−13.3 (− 362.9–409.3)	− 142.5 (− 335.9–0)	182.2 (− 184.5–975.8)	201.3 (− 313.9–636.5)
External_V25Gy_(cc)	−106.5 (− 662.1–123.4)	−16.1 (− 688.9–264.9)	−4.8 (− 410.4–202.4)	−92.1 (− 922–502)	− 292.7 (− 1624.6–163.7)
Non-defined OAR volume (cc)	24,881.5	18,795.5	16,504.1	20,541.4	26,060.8
Non-defined OAR max dose (Gy)	0.2 (−0.4–1.1)	0.1 (− 0.3–0.7)	−0.1 (− 0.9 – 0.5)	0.0 (− 0.6–1.3)	−0.1 (− 1.6–1.2)
Non-defined OAR V10Gy (cc)	56.9 (− 284.5–654.6)	81.2 (− 264.2–461.5)	−46.5 (− 258.6–57.3)	247.9 (− 8.4–931.6)	271.2 (− 208–845.8)
Non-defined OAR V25Gy (cc)	−77.2 (− 615.5–173.8)	3.8 (− 650.3–340.3)	23.1 (− 390–254)	−71.8 (− 888.6–512.8)	−273.9 (− 1617.3–198.4)

**Fig. 2:**
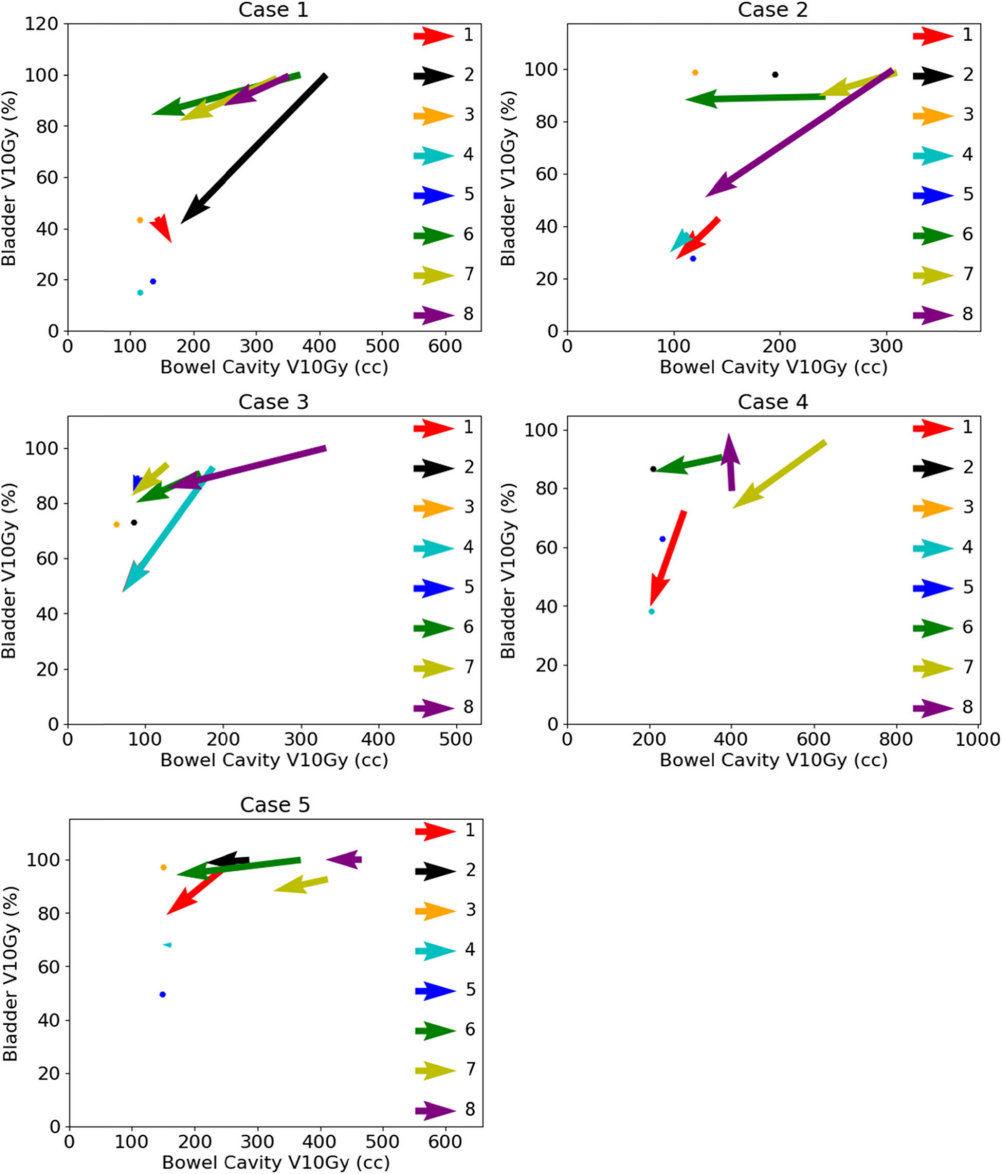
Vector representation for the initial planning and replanning for the bowel cavity V10Gy and the bladder V10Gy for all cases. A vector originates in the values of the DVH parameters of the initial planning and ends in the values of the replanning. The numbers 1 through 8 in the figure legends represent the centers. A plotted point indicates that the corresponding center did not perform replanning

Figure 2 shows for each case the bowel cavity V10Gy and bladder V10Gy for the initial planning and the replanning of all cases. All vectors (except three; center 1 for case 1, center 8 for case 4 and center 4 for case 5) show that both the bowel cavity V10Gy and the bladder V10Gy was lower after replanning. This reduction was at the expense of the V10Gy in the non-defined OAR, which on average is higher after replanning for all cases, except case 3. The bowel cavity V15Gy and bladder V15Gy were lower in 25/30 replanned cases and the bowel cavity V30Gy and bladder V35Gy were lower in 23/30 replanned cases. The bowel cavity V10Gy and V20Gy showed more homogeneity between centers after replanning, as depicted in Fig. 1.

**Fig. 3:**
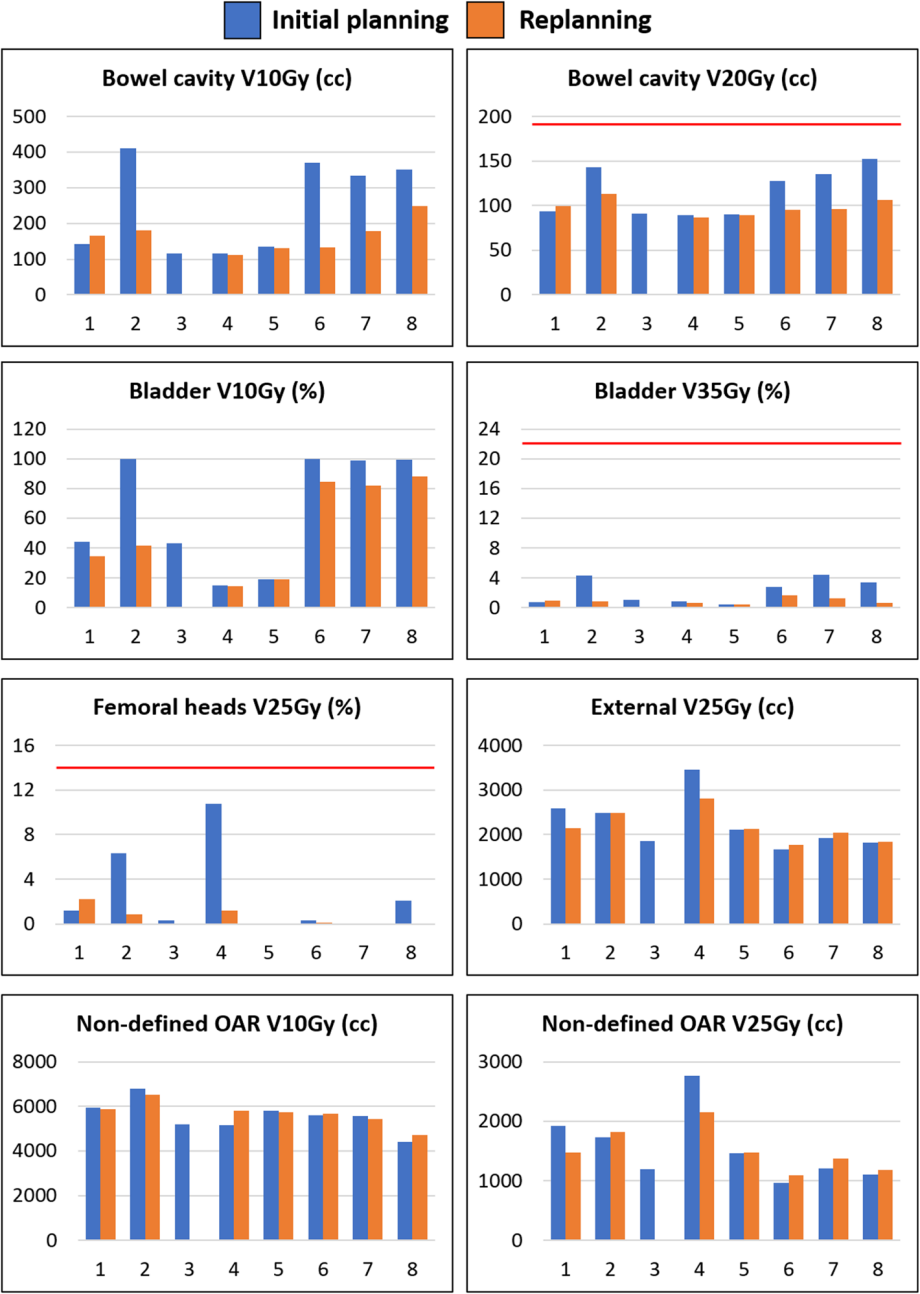
Planning results for the initial planning (blue) and replanning (orange) of case 1. The red lines indicate the OAR optimization objective

**Fig. 4:**
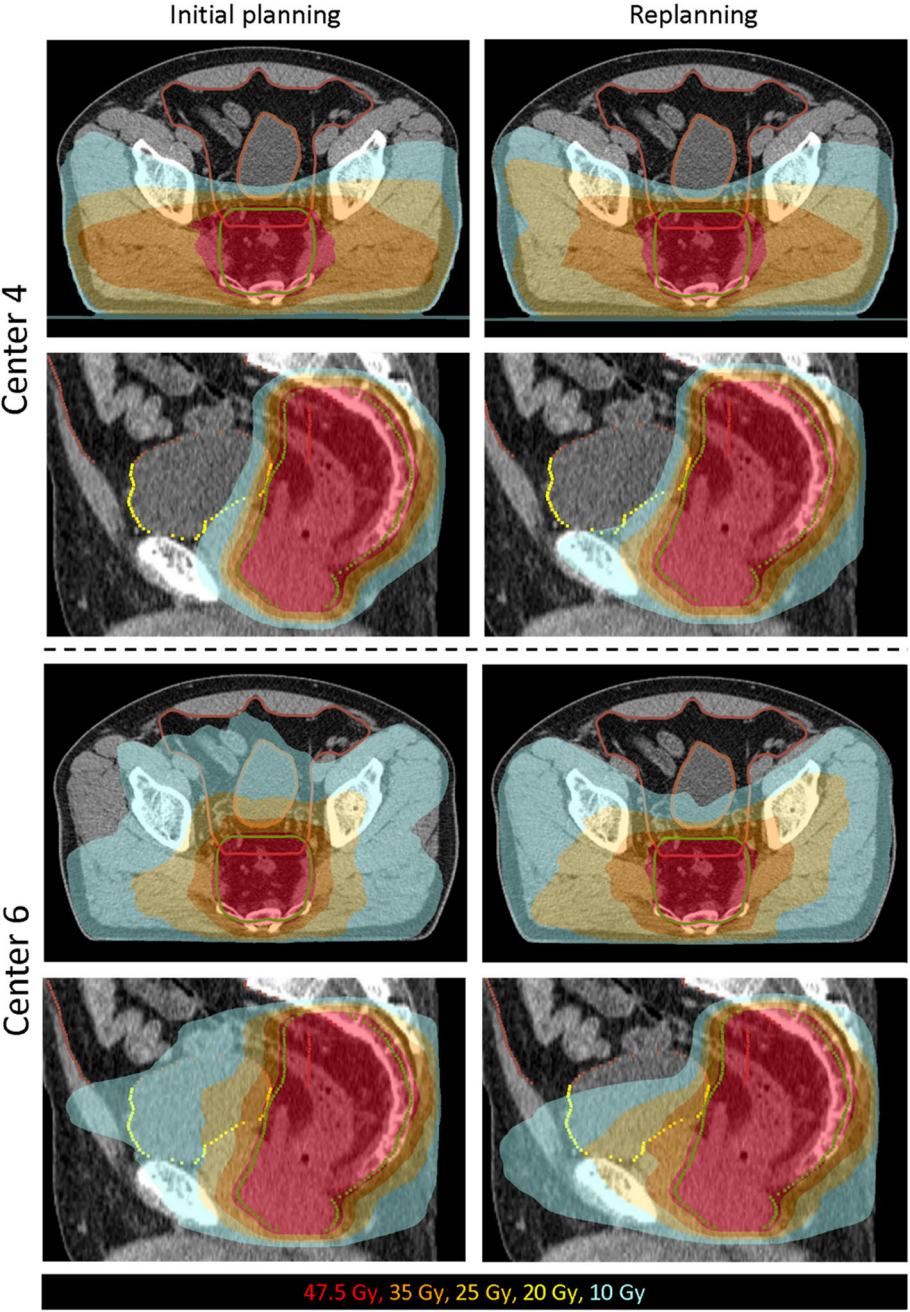
Dose distributions for the initial planning and replanning of case 1 for center 4 and center 6

An example of the initial planning and replanning for one case is shown separately for all centers in Fig. 3. For the same case, the difference in dose distribution between the initial planning and replanning is shown for center 4 and 6 in Fig. 4. For center 4, the replanning reduced the dose deposition laterally, as shown on the axial images, while the V10Gy isodose is expanded into the pubic bone, as shown on the sagittal images. For center 6, replanning reduced the bowel cavity V10Gy and bladder V10Gy while the dose deposition is increased laterally.

## Discussion

The aim of this study was to determine the variability in treatment plans in terms of dose to OAR and to determine the effect of a study group meeting on the quality and variability of treatment plans using a novel target volume for rectal cancer. We have shown that a large variability in dose to OAR occurred while the plans of all centers fulfilled the target constraints. After the study group meeting, replanning resulted in improved treatment plan quality due to lower doses to the defined OAR and smaller differences in dose to OAR between centers.

Optimization objectives for the dose to OAR in the STAR-TReC trial were provided only for dose levels above 20 Gy, which left room for variation in the distribution of the lower dose levels. As a result, radiation-oncologists made different choices regarding the distribution of these lower dose levels or these levels were not taken into account at all during the optimization.

It is not expected that the observed variations in dose to OAR have an impact on the trial hypothesis. However, the observed variations in low dose to the OAR may have an impact on toxicity. Therefore, optimization objectives might be added for the lower dose levels in the upcoming phase III study of the STAR-TReC trial in order to try to reach more consistent and possibly better treatment plans among centers and to prevent unnecessary large volumes of low dose to the OAR. To determine adequate dose volume constraints and prioritization for the OAR in the future, data will be gathered for correlation between dose to OAR and risk of complications within the STAR-TReC trial.

Evaluating a plan on dose volume constraints or objectives alone may not be a good indicator of plan quality, as some patients may have a favorable anatomy and the achieved parameters may not be the lowest possible organ dose volumes. On the other hand, in unfavorable patients, dose may not fulfill planning criteria or objectives while it is still the most optimal plan for that patient. In our study, this can be observed in Fig. 1, where for case 4 the bowel cavity V20Gy approaches the objective for all centers. However, for the other patients the bowel cavity V20Gy is substantially lower than the objective. This shows that careful selection of a benchmark case for trial QA is important and raises the question whether one case is sufficient. Multiple cases enable the evaluation of different patient anatomies with corresponding degrees of possible OAR sparing.

To determine whether the dose to the defined OAR can be lowered further is difficult, even for experienced treatment planners. Plan quality is therefore dependent on planning time, experience of the treatment planner and the degree to which the treatment plan is being critically reviewed. The treatment plans in this study were made and reviewed extensively by expert planners and radiation-oncologists. These treatment plans may therefore not reflect treatment plans produced in clinical practice, as less detailed feedback or discussion of treatment plans is possible and plans may therefore be suboptimal. Automated treatment planning techniques, for example knowledge-based treatment planning, protocol-based automatic iterative optimization or multicriteria optimization, could be used to determine whether a treatment plan can be further optimized [14].

Other studies describing trial QA report on the identification of delineation and/or dosimetric violations and that participating centers receive individual feedback regarding those violations [15–17]. Subsequently, the violations were resolved and treatment plan quality was improved. In our study, however, all target constraints and OAR objectives were fulfilled and the question was how to handle the variations in dose distribution for the OAR for which no guidelines were yet available. The study group meeting has enabled us to discuss the planning results and the considerations regarding dose distribution to defined and non-defined OAR face-to-face, by doing so learning from each other. Furthermore, sharing the planning results of the initial planning of all centers enabled centers to compare their results and helped them decide whether further optimization of the treatment plan was possible and desired for each case. Consequently, replanning led to improved plan quality as lower doses to defined OAR were observed while maintaining target volume constraints. Importantly, although no consensus guidelines were made on how to handle the variations in dose to OAR, the variation of dose levels below 20 Gy was reduced after replanning.

## Conclusions

In the STAR-TReC trial, no constraints or objectives are defined for the OAR for lower dose levels since there is no clinical evidence to base constraints on. As a result, in this treatment planning study the dose to OAR varied considerably between centers, especially for dose levels below 20 Gy. After the study group meeting, treatment plan quality was improved as replanning resulted in lower dose to the defined OAR and reduced variability between centers. Therefore, for a novel target volume, we recommend to include more than one QA case and to share all planning results with participating centers, possibly at a study group meeting, to allow them to compare results and decide whether further optimization is possible.

## Data Availability

The datasets generated and/or analyzed during the current study are not publicly available because the associated trial is still open and running.

## Abbreviations

CI: Conformity Index
CTV: Clinical Target Volume
LC-CRT: Long-course Chemoradiotherapy
OAR: Organs At Risk
PTV: Planning Target Volume
QA: Quality Assurance
SC-RT: Short-course Radiotherapy
TME: Total Mesorectal Excision
